# RNA sequencing and anthocyanin synthesis-related genes expression analyses in white-fruited *Vaccinium uliginosum*

**DOI:** 10.1186/s12864-018-5351-0

**Published:** 2018-12-13

**Authors:** Yang Yang, Baihui Cui, Zhiwen Tan, Bingxue Song, Hounan Cao, Chengwen Zong

**Affiliations:** Agriculture College of YanBian University, Yanji, Jilin, 133002 China

**Keywords:** *Vaccinium uliginosum*, White-fruited variant, RNA-seq, qRT-PCR

## Abstract

**Background:**

*Vaccinium uliginosum* (Ericaceae) is an important wild berry having high economic value. The white-fruited *V. uliginosum* variety found in the wild lacks anthocyanin and bears silvery white fruits. Hence, it is a good resource for investigating the mechanism of fruit color development. This study aimed to verify the differences in the expression levels of some structural genes and transcription factors affecting the anthocyanin biosynthesis pathway by conducting high-throughput transcriptome sequencing and real-time PCR analysis by using the ripening fruits of *V. uliginosum* and the white-fruited variety.

**Results:**

We annotated 42,837 unigenes. Of the 325 differentially expressed genes, 41 were up-regulated and 284 were down-regulated. Further, 11 structural genes of the flavonoid pathway were up-regulated, whereas two were down-regulated. Of the seven genes encoding transcription factors, five were up-regulated and two were down-regulated. The structural genes *VuCHS*, *VuF3’H*, *VuFHT*, *VuDFR*, *VuANS*, *VuANR*, and *VuUFGT* and the transcription factors *VubHLH92*, *VuMYB6*, *VuMYBPA1*, *VuMYB11*, and *VuMYB12* were significantly down-regulated. However, the expression of only *VuMYB6* and *VuMYBPA1* rapidly increased during the last two stages of *V. uliginosum* when the fruit was ripening, consistent with anthocyanin accumulation.

**Conclusions:**

*VuMYB6* was annotated as *MYB1* by the BLAST tool. Thus, the white fruit color in the *V. uliginosum* variant can be attributed to the down-regulation of transcription factors *VuMYB1* and *VuMYBPA1*, which leads to the down-regulation of structural genes associated with the anthocyanin synthesis pathway.

**Electronic supplementary material:**

The online version of this article (10.1186/s12864-018-5351-0) contains supplementary material, which is available to authorized users.

## Background

*Vaccinium uliginosum* is a perennial deciduous shrub of the Ericaceae family. It is distributed in the northeast regions of China, including Xiao Xing’an Mountains, Da Xing’an Mountains, Inner Mongolia, and Changbai Mountain forest area, it also occurs in Sphagnum swamp meadow at an elevation of more than 700 m, along with a large community of other plant species such as *Carex*, *Larix gmelinii*, *Betula ovalifolia*, and *Ledum palustre*. This species can be found at temperatures as low as − 40 °C to 50 °C [1]. The berries are fragrant, delicious, and nutritious and can be consumed either raw or after processing [2]. Although *V. uliginosum* is a very valuable species, it has not been intensively investigated. *V. uliginosum* berries contain abundant amino acids, trace elements, anthocyanins, procyanidins and other polyphenols [3]. They have various benifical health effects such as blood vessels softening, disease prevention and health care.

**Fig. 1:**
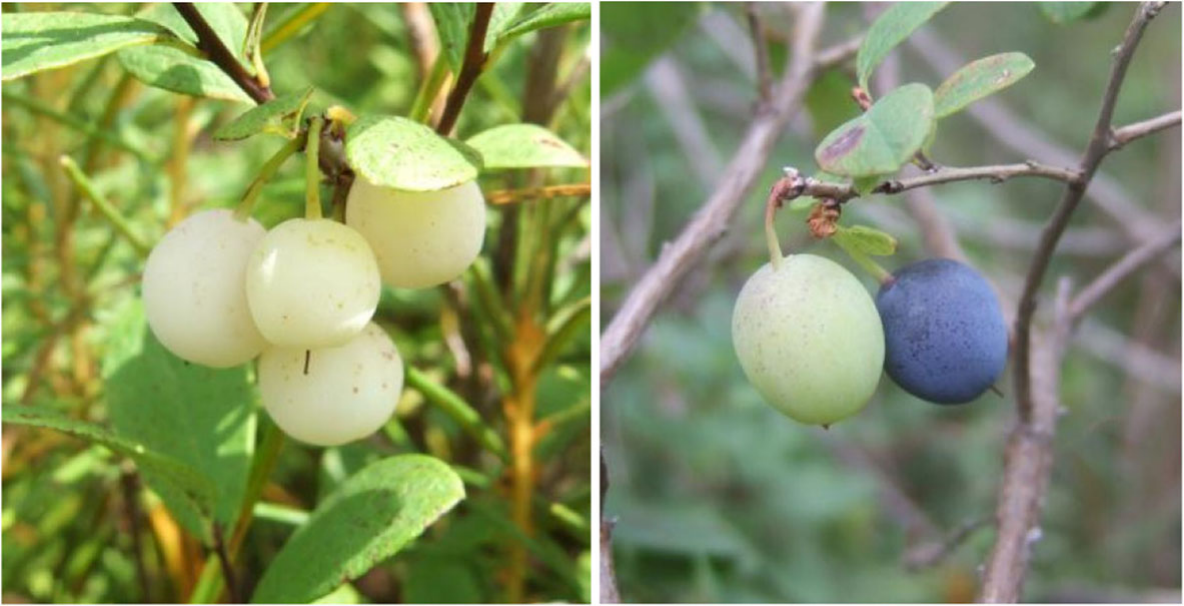
The ripening white *Vaccinium uliginosum* variety and the chimera at the experimental site. The site is in Wangqing County of Jilin Province Lanjia forest farm

A *V. uliginosum* variety with white berries has been reported, but no further studies on this species were performed [4, 5]. During resource investigation, we found a white-fruited *V. uliginosum* variety in Wangqing Country of Jilin Province in the Lanjia forest farm. They were sporadically distributed in the wild community. The berries were round or oblong, and the fruit weight was approximately 1–1.5 g, the ripening berries were silvery white and translucent. Preliminary investigation revealed that the berries lacked anthocyanins, and their total phenolic and flavonoid content was lower [6], whereas the vitamin C and titratable acid contents were higher than those of the wild-type *V. uliginosum*. During the survey, we also found a chimera with blue and white berries on the same plant during the survey (Fig. 1). This phenotypic variation might have been caused by somatic mutation. This resource has a potential value in theoretical research and application for elucidating the mechanism of the synthesis and regulation of anthocyanidin. The white-fruited *V. uliginosum* variety produces tastier and sweeter berries with low tannin content than those of the wild type. This might reduce the processing and marketing cost of the berries [5].

Anthocyanin is a flavonoid produced via the flavonoid biosynthesis pathway during plant metabolism. It is an important water-soluble, nature, edible pigment, which exists widely in the vacuoles of plant epidermal cells and imparts orange and red to blue color to flowers, fruits, stems, leaves, and toots [7]. It is safe, non-toxic, and abundant, with important nutritional and pharmacological effects, such as antioxidant capacity, and ability to scavenge free radicals, prevent cardiovascular diseases, tumors, mutation, and radiation, regulate the activity of platelets, prevent platelet condensation, and induce immunomodulatory activity. In addition, it helps in improving the cold and drought resistance ability of plants [8].

The anthocyanin biosynthetic pathway is most extensively studied secondary metabolic pathway in plants studied most extensively, particularly in *Arabidopsis*, *Zea* and *Petunia*. Anthocyanin biosynthesis can be divided into two stages. First, phenylalanine transforms into 4-coumaryl: CoA referred to as the phenylpropanoid metabolic pathway. Subsequently, 4-coumaryl: CoA transforms into all kinds of flavonoid compounds, which is referred to as the anthocyanin biosynthetic pathway. Anthocyanin biosynthesis involves various structural and regulatory genes [9] .

The genes involved in anthocyanin biosynthesis can be classified into two major groups. The first group consists of structural genes that directly encode the key enzymes in anthocyanin biosynthesis. The other group is transcriptional factors. Transcriptional regulation is an important aspect of the regulation of gene expression in the anthocyanin biosynthetic pathway of plant. Its mechanism is very complicated. At present, three main types of regulatory factors have been identified: MYB, bHLH and WD40 transcription factors [10]. Anthocyanin biosynthesis in most of the species is regulated by the transcription factors that form a protein complex that binds to the promoter of the structural genes. The MADS-box transcription factor *VmTDR4* in *V. myrtillus* was suggested to play an important role in the synthesis of anthocyanins by the direct or indirect regulation of MYB transcription factors [11]. Moreover, the microRNA miR156 and its target gene *SPL3* decrease anthocyanin biosynthesis in *Arabidopsis thaliana* [12].

Fruit color is an important factor affecting the appearance and quality of the fruit. The study of the mechanism and regulation of fruit coloration is very important for elucidating somatic cell mutations in the peel. Mutations affecting fruit coloration, have been investigated in the model plants such as grape [13, 14], *V. myrtillus* [15], *Duchesnea indica* [16], and *Syzygium malaccense* [17]. The variations in the regulatory or structural genes in the anthocyanin biosynthesis pathway of these species are responsible for the mutations.

However, few studies have investigated the variations in the molecular mechanism of anthocyanin biosynthesis in *V. uliginosum*. This study aimed to analyze the differences among wild *V. uliginosum* and fruit color varieties at the molecular level by using transcriptome sequencing analysis. In addition we conducted a bioinformatics analysis and quantitative polymerase chain reaction (q-PCR) to identify the genes related to anthocyanin biosynthesis pathway and glucose metabolism. Our findings might reveal the main reason for the lack of anthocyanins in the *V. uliginosum* mutant.

## Results

### Transcriptome sequencing and assembly

In order to investigate the mutation mechanism of the white-fruited variant, we repeated the transcriptome sequencing analysis thrice. We obtained 34.62Gb of Clean Data, which reached the 5.3Gb threshold for each sample and Q30 base percentage was ≥88.73%. The Read Number was more than 21,044,015, and the base number for each sample was not less than 5,303,019,780. The *V. uliginosum* GC content was in the range of 46.69—47.17%, whereas that of the white-fruited variant was in the range of 47.17—47.46% (Table 1).

**Table 1:** Statistical evaluation of sequencing data

Samples	Read Number	Base Number	GC Content	Q30 (≥%)
A1	21,137,847	5,326,737,444	46.78%	88.73%
A2	23,116,662	5,825,398,824	46.69%	90.89%
A3	25,635,645	6,460,182,540	47.17%	90.61%
B1	24,328,728	6,130,839,456	47.36%	90.85%
B2	22,137,140	5,578,559,280	47.46%	90.61%
B3	21,044,015	5,303,091,780	47.17%	90.97%

Note: A1, A2, A3 is the sample of *Vaccinium uliginosum*. B1, B2, B3 is the sample of its white-fruited variant

The assembled sequences showed 225,777,919 transcripts items and 89,725 unigenes. The N50 value of the transcripts and unigenes were 1058.35 and 1094, respectively. This showed that the assembly had high integrity (Table 2). The number of contigs with 200—300 nt was the highest (27,838,434; 99.62%) and that of contigs with length of > 2000 nt was the lowest (6441; 0.02%). In addition, the highest number of transcripts had length in the range of 200—300 nt, and the lowest number of transcripts had 1000—2000 nt (27,320; 12.81%). However, no unigenes had length in the range of 200—300 nt and the highest number of unigenes had length of 300—500 nt (42,605 accounting for 47.48% of the total).

**Table 2:** Assembly results

Length Range	Contig	Transcript	Unigene
200–300	27,732,934(99.62%)*	68,779(32.24%)	0%
300–500	52,388(0.19%)	65,549(30.73%)	42,605(47.48%)
500–1000	32,032(0.12%)	51,682(24.23%)	26,647(29.70%)
1000–2000	14,639(0.05%)	27,320(12.81%)	13,722(15.29%)
2000+	6441(0.02%)	213,330	6751(7.52%)
Total Number	27,838,434	225,777,919	89,725
Total Length	1,139,232,020	1516	73,630,818
N50 Length	43	1058.35	1094
Mean Length	40.92		820.63

Analysis by sequence alignment revealed approximately 18,355,782 mapped reads in *V. uliginosum*, accounting for 78.77% of the total clean reads. The number of mapped reads of the white-fruited variant was approximately 17,688,233 (78.58%). The mapped reads were not significantly different between the two samples (Table 3).

**Table 3:** Comparison of the results of sequencing and assembly

Number	Clean Reads	Mapped Reads	Mapped Ratio
A1	21,137,847	16,607,615	78.57%
A2	23,116,662	18,181,365	78.65%
A3	25,635,645	20,278,365	79.10%
B1	24,328,728	19,199,505	78.92%
B2	22,137,140	17,420,598	78.69%
B3	21,044,015	16,444,595	78.14%

### Functional annotation of unigenes

A total of 89,725 unigenes were annotated from different databases, with a total of 42,837 unigenes with functional annotations. Unigenes with a length of 300—1000 nt and ≥ 1000 nt were 26,734 (62.41%) and 16,103 (37.59%), respectively. Analysis by sequence alignment revealed that the maximum number of unigenes were annotated in NR (40,121, accounting for 93.66% of all the annotations). Whereas, the least number were annotated in COG (12,991, accounting for 30.33% of the total). The remaining unigenes were annotated in GO, KEGG, KOG, Pfam, and Swiss-prot databases (Table 4).

**Table 4:** Unigene annotation in different databases

Anno_Database	Annotated_Number	300 ≤ length ≤ 1000	Length ≥ 1000
COG_Annotation	12,991	6985	6006
GO_Annotation	24,469	15,061	9408
KEGG_Annotation	17,732	11,297	6435
KOG_Annotation	25,080	15,162	9918
Pfam_Annotation	29,299	15,609	13,690
Swissprot_Annotation	27,560	16,170	11,390
nr_Annotation	40,121	25,211	14,910
All_Annotated	42,837	26,734	16,103

### Screening of differentially expressed genes

For differential expression analysis, we corrected the significance value of the original hypothesis of the accepted effective Benjamini—Hochberg method. Finally, we adopted the corrected *P* value named false discovery rate (FDR) as the key indicator of differential gene expression, which can help to reduce the false positive rate by considering the expression levels of numerous of genes in an independent statistical hypothesis test.

**Fig. 2:**
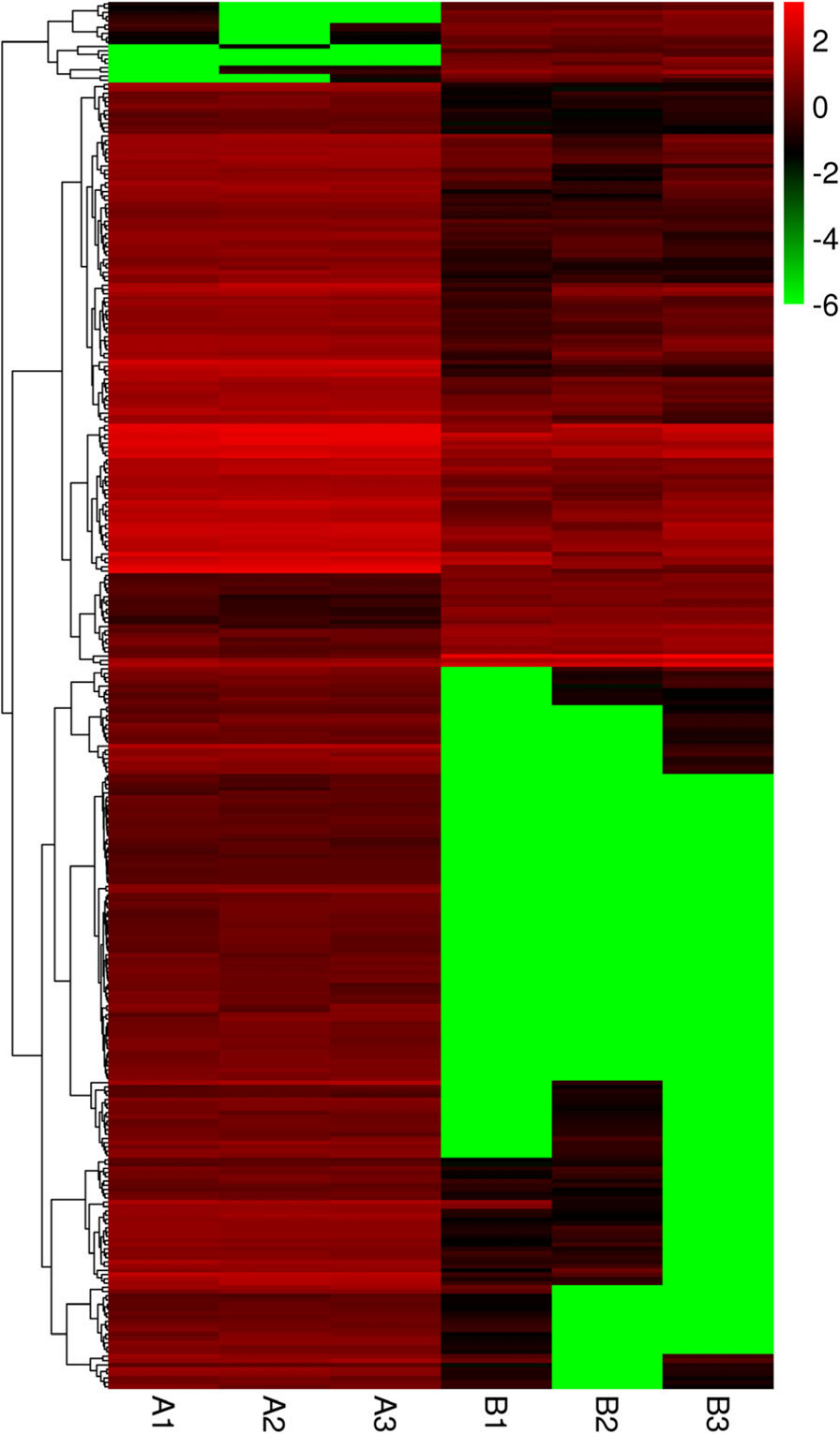
Differential expression gene expression pattern clustering map**.** A1, A2, A3: sample of *V. uliginosum* ripening fruits. B1, B2, B3: sample of white-fruited variant ripening fruits

A total of 325 differentially expressed genes (DEGs) were obtained, among which 41 were up-regulated and 284 genes were down-regulated (Fig. 2). A total of 118,804 genes showed no significant differences in expression level.

Of the 41 genes of up-regulated genes, 28 and 13 DEGs annotated and unannotated, respectively. Unannotated genes might be the new transcripts of *V. uliginosum*. The expression level of *F3’5’H* (c101538) and *DFR* (c130571) genes of the flavonoid biosynthesis pathway was high (6.1665 and 2.5403, respectively). The c74288 gene expressed in the white-fruited variant, but not in *V. uliginosum*; this could be attributed to the difference in aromatic compound metabolism (Additional file 1: Table S1).

Of the 284 down-regulated genes, 148 and 136 DEGs were annotated and unannotated, respectively. Moreover, 71 genes were expressed in *V. uliginosum*, but not in the white-fruited variant. The c17593 gene related to transcription factor B3, c114838 gene related to MADs-box transcription factor, and c86331 gene related to transcription factor *MYB3* were down-regulated in the white-fruited variant, with expression fold values of − 5.1825, − 4.7228, and − 6.6772, respectively. The gene c89580 related to sugar metabolism was not expressed in the white-fruited variant, but was expressed in *V. uliginosum*. In addition, the c116690 gene was down-regulated in the white-fruited variant and its expression fold value was − 2.9947 (Additional file 2: Table S2).

### Functional annotation of DEGs

In all, 176 annotated DEGs were different between *V. uliginosum* and its white-fruit variant among the different databases. In the NR database, a maximum of 159 DEGs were annotated. In addition, a minimum of 39 DEGs were annotated in the COG database (Table 5).

**Table 5:** The number of annotated differential expression genes

DEG Set	Annotated	COG	GO	KEGG	KOG	Pfam	Swiss-Prot	nr
A vs B	176	39	82	50	94	145	114	159

### GO function enrichment of DEGs

**Fig. 3:**
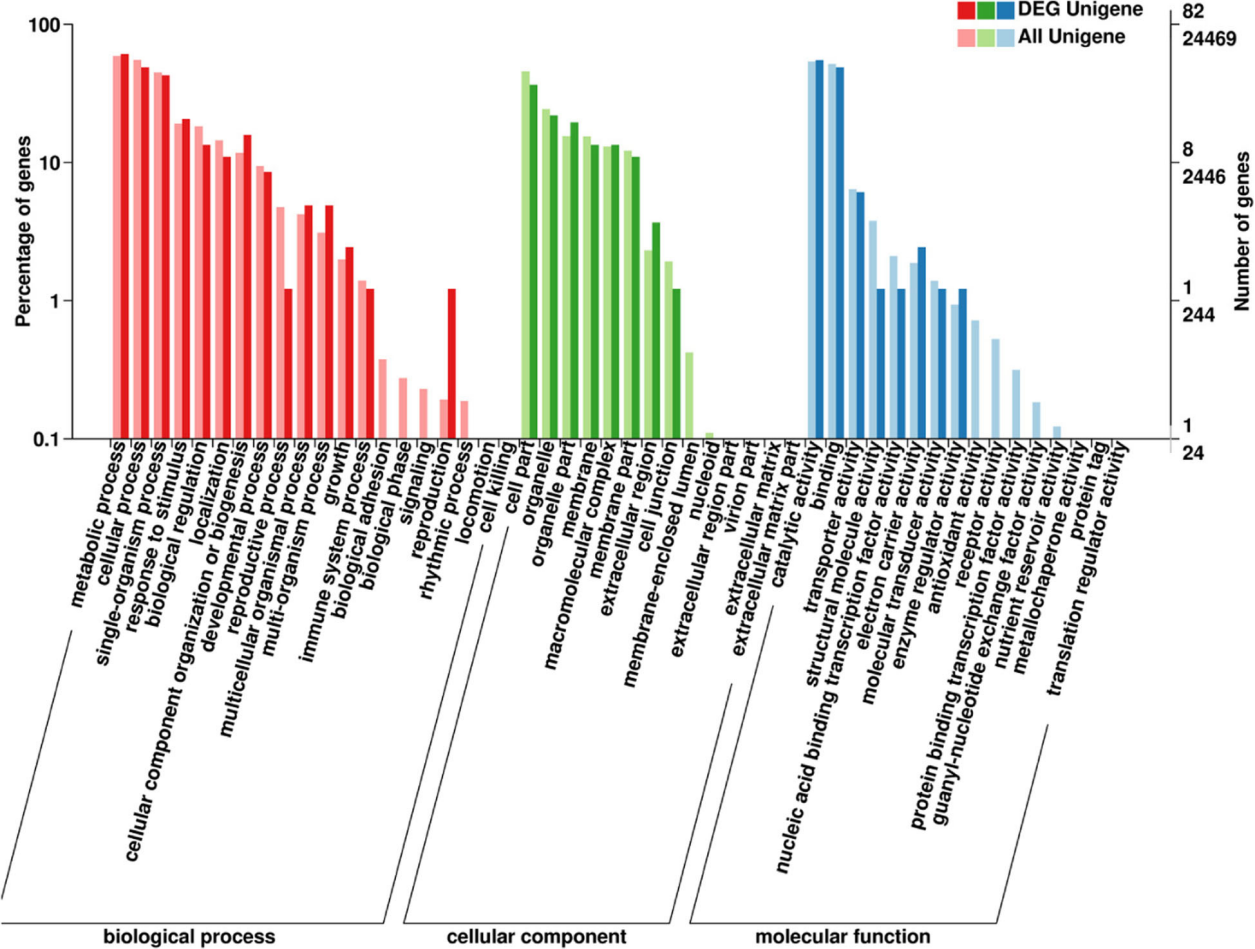
The secondary node of GO annotation statistical figure of differentially expressed genes

GO function enrichment revealed 82 annotated DEGs (Fig. 3). They participate in three major aspects including the biological process, molecular function and cellular components. In all, 88 GO terms were enriched. Of them, 47 were significantly enriched (*P* ≤ 0. 05). Moreover GO: 0009773 was the most significantly associated (*P* = 0.0002) with the light system I photoelectron transfer. The GO: 0009058 node was related to the biosynthesis process, and two DEGs were associated with this node one is sucrose synthase (c122231), the FPKM value of which was four fold lower in *V. uliginosum* and the other is c134490 of the glycosyl transferase group which was not expressed in the white-fruited variant. However, its FPKM value was 10.7933 in *V. uliginosum* (Additional file 3: Table S3).

### Enrichment analysis of DEGs in the KEGG pathway

In living organisms, different gene products coordinate to perform different biological functions. Pathway annotation analysis of DEGs is helpful for further understanding the function of genes. KEGG database is the main public database on pathways. We analyzed of whether the appearance of DEGs in a pathway reflected pathway enrichment. KEGG pathway enrichment analysis revealed 42 annotated DEGs and 15 pathways. These pathways involve five aspects, including cellular processes, genetic information processing, human diseases, metabolism, and organismal systems.

Among them, the number of unigenes and DEGs involved in the phenylpropanoid biosynthesis pathway was 240 and 5, respectively. The flavonoid biosynthesis pathway had 80 unigenes, and three DEGs. In addition, the numbers of unigenes and DEGs in starch and sucrose metabolism pathways were 390 and two, respectively (Additional file 4: Table S4).

### Anthocyanin synthesis-related genes expression

**Fig. 4:**
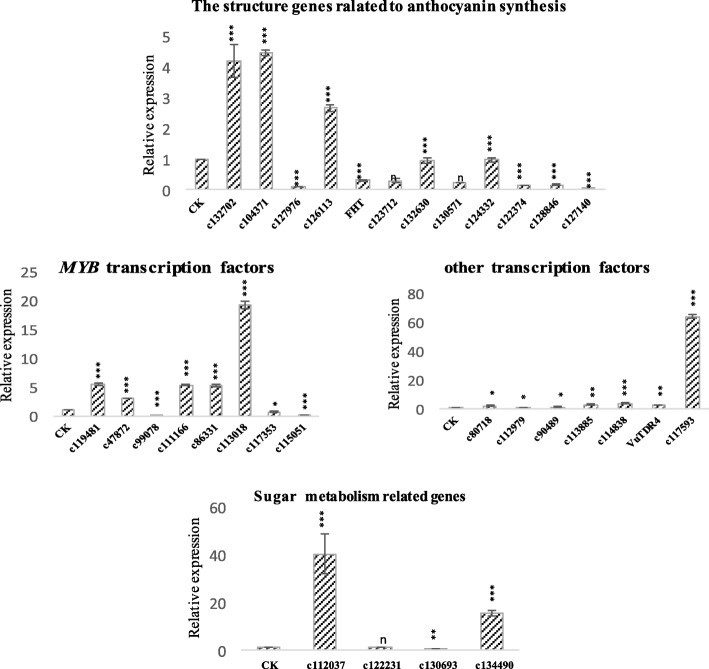
Relative expression of the genes of *V. uliginosum* and its white-fruited variant**.** Values represent mean ± SD of three replicates. *P ≤ 0. 05, **P ≤ 0. 01, ***P ≤ 0. 001, and “n” is not statistically significant difference (independent samples *t*-test) Note: CK: *V. uliginosum* ripening fruits related genes expression. The other is related genes are expressed in white-fruited variant ripening fruits

The color of fruit peel is known to be correlated with the expression of anthocyanin synthesis related genes. Our expression analysis results showed that the expression of structural genes was mostly down-regulated in the white-fruited variant. However the expression of transcription factors in the anthocyanin biosynthesis pathway and genes in the sugar metabolism pathway were mostly up-regulated (Fig. 4). The structural genes peroxidase (*VuPOD*, c132702), cinnamoyl-CoA reductase (*VuCCR*, c104371), and *VuCHI* (c126113) related to anthocyanin biosynthesis were significantly up-regulated in the white-fruited variety. In contrast, the genes *VuCHS* (c127976), flavanone-3β-hydroxylase (*VuFHT*), flavonoid 3′-hydroxylase (*VuF3’H*, c123712), *VuDFR* (c130571), *VuANS* (c122374), anthocyanidin reductase (*VuANR*, c128846), and *VuUFGT* (c127140) were significantly down-regulated. However, the expression of flavonoid3’, 5’hydroxylase (*VuF3’5’H*, c132630) and leucoanthocyanins reductase (*VuLAR*, c124332) was not significantly different between the white-fruited variant and wild-type *V. uliginosum*. The transcription factors were in the most up-regulated in the white-fruited variant. The genes *VubHLH63* (c80718), and *VuTDR4* and transcription factors *VubHLH130* (c113885), *VuMADS-box* (c114838) and transcription factor B3 (c117593) were significantly up-regulated in the white-fruited variant. However the expression of *VubHLH93* (c90489) was not significantly different from that of *V. uliginosum*. In the white-fruited variant, the *VubHLH92* (c112979) gene was significantly down-regulated. Among the MYB transcription factors, *VuMYB2* (c119481), *VuMYB4* (c47872), *VuMYB7* (c111166), *VuMYB8* (c86331), and *VuMYB10* (c113018) were significantly up-regulated. The transcription factors *VuMYB6* (c99078) and *VuMYBPA1* (c115051) were significantly down-regulated. Moreover, the *VuMYB12* (c117353) transcription factor was significantly down-regulated. In the white-fruited variant sugar metabolism, beta-glucosidase (c112037) and glycosyl transferases group 1 (c134490) were significantly up-regulated, and the sugar (and other) transporter (c130693) was significantly down-regulated. The sucrose synthase (c122231) expression was not significantly different from that of *V. uliginosum*.

### The expression of differentially expressed transcription factors at different developmental stages in *V. uliginosum*

Four transcription factors, *VubHLH92* (c112979), *VuMYB6* (c99078), *VuMYBPA1* (c115051), and *VuMYB12* (c117353), were expressed in the white-fruited variant, but significantly down-regulated in *V. uliginosum*.

**Fig. 5:**
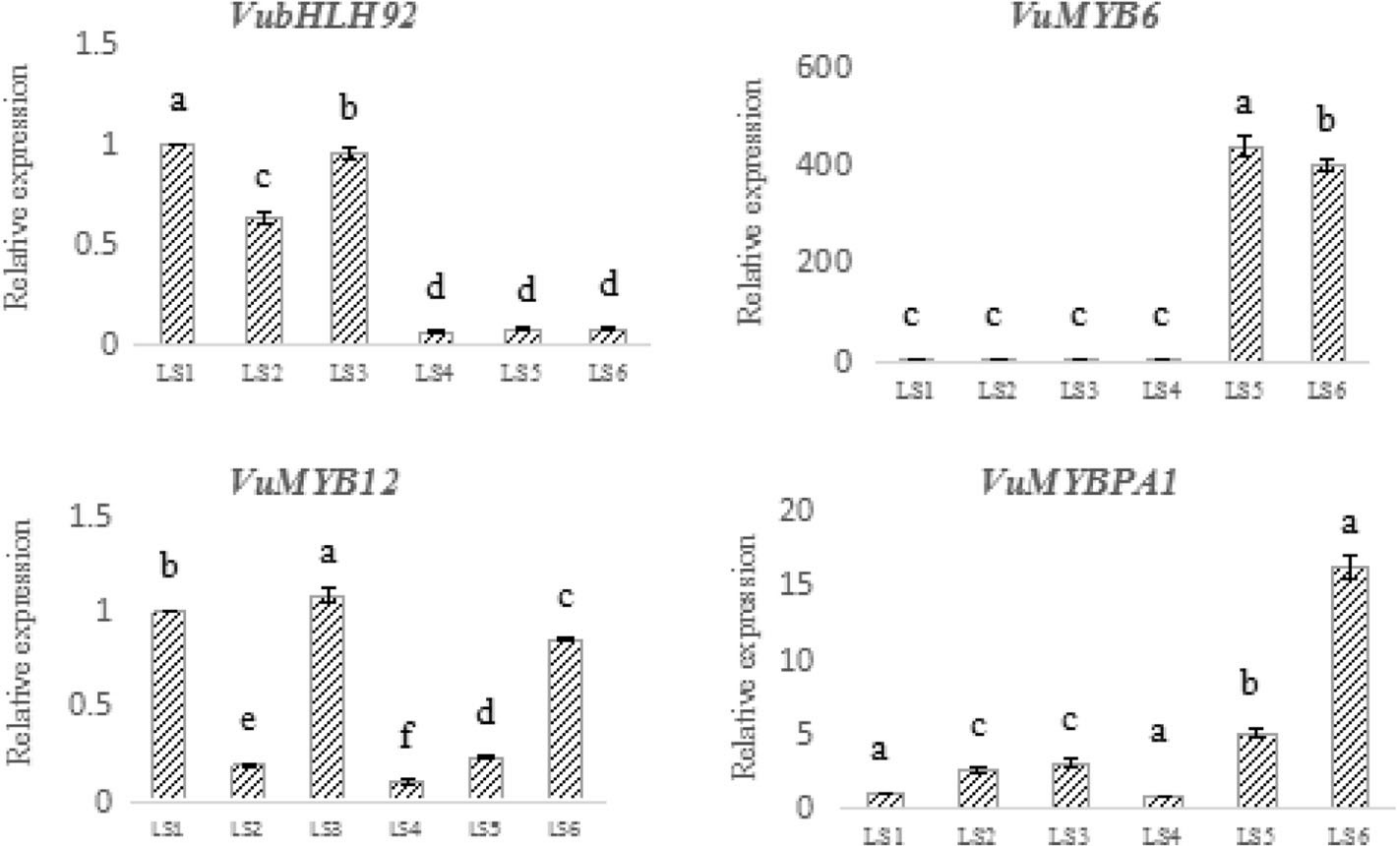
The differential expression of transcription factors in *V. uliginosum* at the six developmental stages. The column represent means ± SD from three independent biological replicates. Statistical analysis was performed using Duncan test at the level of *P* ≤ 0. 05

Analysis of the expression of these four transcription factors at different developmental stages of *V. uliginosum* showed that the expression of *VubHLH92* was first decreased and then increased (Fig. 5). When the fruit was about to ripen, almost no *VubHLH92* expression was noded. The expression of the *VuMYB6* was almost the same during the first four stages, and then the expression suddenly and rapidly increased when the fruit was ripening, to approximately 400 times of that during the first period, consistent with the accumulation patten of anthocyanin. Evidently, this transcription factor is related to the synthesis of anthocyanin in *V. uliginosum*. The *VuMYB12* transcription factor expression was first decreased and then increased, followed by a decline. The expression began to increase when the fruit was ripening, but its expression was lower than that during the first and third periods. The expression of *VuMYBPA1* transcription factor first increased gradually and decreased in the fourth period, and increased rapidly in the last two periods. The expression ratio of the last period was approximately 16 times higher than that for the first period, consistent with the accumulation of anthocyanin.

## Discussion

As early as 1986, Zhang et al. [5] identified a variant of *V. uliginosum* in Heihe City, Heilongjiang Province. They found that the sugar content of the white-fruited variety was higher than that of the blue-fruited wild-type one. Moreover, Ma et al. [4] reported that the white-fruited variant was slightly sweeter than the original variety from the Da Xing’an Mountains of China. These findings might explain why most of the genes related to sugar metabolism were up-regulated in the white-fruited variant. The expression of beta-glucosidase (c112037) was significantly up-regulated, probably because glucose was the principle sugar in the fruits of the white-fruited variant. Glycosyltransferase is the key enzyme associated with the catalytic synthesis of sugar chains; thus, the extremely significant up-regulation of glycosyl transferases group 1 (c134490) was reasonable. Moreover the sucrose is not primarily responsible for the sweetness of the white-fruited variety; thus, sucrose synthase (c122231) expression was not significantly different from that in *V. uliginosum*. After sugar formation in ripe fruits, the expression of sugar (and other) transporter (c130693) was significantly down-regulated in the white-fruited variant.

According to our survey, the morphological characteristics of the white-fruited variant did not differ from those of *V. uliginosum*, except that the ripe fruit was silver-white and lacked anthocyanin. This is not caused by the external environment, as the silver-white coloration does not change even in winter [4]. The formation of fruit peel color is closely related to the synthesis of anthocyanins, and its mechanism is complex. The expression of structural genes in the anthocyanin biosynthetic pathway is generally required for upstream transcription factor binding to initiate gene transcription and expression, to finally synthesize anthocyanins and impart color to the fruit and peel [18].

The expression of structural genes *VuF3’ 5’ H* (c132630) and *VuLAR* (c124332) of the anthocyanin biosynthesis pathway in the white-fruited variant did not differ significantly from that in *V. uliginosum*. These genes do not direct effects on anthocyanin synthesis. The expression of *VuCHS* (c127976), *VuFHT*, *VuF3’H* (c123712), *VuDFR* (c130571), *VuANS* (c122374), *VuANR* (c128846) and *VuUFGT* (c127140) genes was extremely significantly down-regulated in the white-fruited variety their expression is closely linked to anthocyanin synthesis. This is probably because of the inhibition of transcription factor expression, which can affect the accumulation of anthocyanin.

Transcription factor B3 is unique to plants; it is a super-family existing mainly in gymnosperms, mosses, and algae and other plants. Luo et al. found that this transcription factor B3 plays an important role in the plant stress response and in plant growth and development [19]. Zhang et al. [20] found that transcription factor B3 plays a key role in the development and maturation of the embryo in *Theobroma cacao*. In *Arabidopsis*, the excessive expression of the transcription factor B3 resulted in the loss of petals [21]. At present, no studies have shown a relationship between transcription factor B3 and coloring. The expression of transcription factor B3 in the white-fruited variant was extremely significantly up-regulated; therefore, it might play a role in other aspects of development.

The *MADS-box* and *TDR4* genes encode transcription factors, and the *TDR4* gene is a member of the MADS gene family [11]. Previous studies have showed that *MADS* and *TDR4* genes were involved in plant anthocyanin synthesis. Another study showed that *IbMADS10* was involved in the synthesis of anthocyanin in *Ipomoea batatas* [22]. In the *V. myrtillus* mutant, down-regulated expression of the *VmTDR4* gene directly or indirectly inhibited the expression of the *VmMYB2* gene, resulting in the down-regulation of the structural genes of the anthocyanin biosynthesis pathway, inhibiting the synthesis of anthocyanin [11]. The MADS super family has many members, and each member has its own function. We verified that the expression of the *VuMADS-box* (c114838) gene was extremely significantly up-regulated and that of the *VuTDR4* gene was significantly up-regulated in the white-fruited variety. Therefore, they might have no relation with anthocyanin synthesis.

At present, studies on anthocyanin synthesis in plants suggest that the known transcription factors can be mainly divided into three categories, namely the R2R3-MYB type, bHLH and WD40 complex protein transcription factors [10]. Some of these are single gene regulatory structural genes, some are two genes whose interaction controls anthocyanin synthesis, and some are MBW (MYB bHLH WD40) complexes regulating anthocyanin accumulation. Park et al. [23] revealed that the *bHLH2* gene contained a DNA transposon resulting in a change in the color of the petals of *Pharbitis purpurea* flowers. A single gene mutation can also affect anthocyanin accumulation. Qian et al. [24] found that *PyMYB10* regulated the synthesis and expression of anthocyanin in *Pyrus pyrifolia*. Azuma et al. [25] confirmed that *ViMYBA1–3* is a key gene that influences anthocyanin biosynthesis in grape peel. Aharoni et al. [26] repored that yellow strawberry *FaMYB1* inhibited the accumulation of anthocyanin and flavonoids in transgenic tobacco. Primetta et al. [27] showed that the homologous *MYBPA1* and *MYB2* are the members of the R2R3MYB family, and their down-regulation inhibited the expression of *CHS*, *DFR*, *LAR*, *ANR*, *ANS* and *UFGT* structural genes, affecting the accumulation of anthocyanin. Single-gene transcription factor can also directly regulate anthocyanin synthesis. Wada et al. [28] reported that *Arabidopsis* MYB (*CPC*) and bHLH (*GL3*) interact with each other to influence bHLH anthocyanin pigment synthesis in *Lycopersicon esculentum*. Schwinn et al. [29] reported that MYB and bHLH transcription factors increased the intensity of the color of *Petunia* and *Platycodon*, and strongly enhanced the phenotype of *Petunia*. Liu et al. [30] reported that *MrbHLH1* and *MrMYB1* affected anthocyanin synthesis in tobacco and *Myrica rubra*. Two transcription factors can also interact to regulate the accumulation of anthocyanin. Xie et al. [31] showed that *MdbHLH3* regulated *MdMYB1* expression to mediate the low-temperature-induced anthocyanin accumulation and coloration of apples. A transcription factor can also be regulated by another transcription factor to affect the biosynthesis of anthocyanin. Albert et al. [32] confirmed that the MBW complex transcription factor activated a single transcription factor and then the R2R3-MYB type transcription factors *TrMYB133* and *RrMYB134* regulated the biosynthesis of anthocyanin and procyanidin in *Trifolium repens*. Thus, a single transcription factor can thus be regulated by a complex of transcription factors to induce the synthesis of anthocyanin.

Primetta et al. [27] used *V. uliginosum* and the white-fruited variant from Finland as study material and indicated that the expression of structural genes *CHS*, *DFR*, *LAR*, *ANR*, *ANS* and *UFGT*, related to the anthocyanin synthesis pathway, was significantly down-regulated, and the expression of *CHI* and *F3’5’H* genes did not differ significantly from those in *V. uliginosum*. However, in our study, the expression of *VuCHS*, *VuFHT*, *VuF3’H*, *VuDFR*, *VuANS*, and *VuUFGT* was significantly down-regulated in the white-fruited variety, and the expression of the *VuF3’ 5’ H* and *VuLAR* genes did not differ significantly from that in *V. uliginosum*. Nevertheless, the expression of *VuCHI* was significantly up-regulated. The results of our study and those of Primetta et al. differ to some extent owing to the fact that mutants are formed differently because of the difference geographical regions.

In this study, high-throughput transcriptome sequencing was used to identify DEGs, the expression of which was verified to differ significantly between wild-type *V. uliginosum* and the white-fruited variant using qRT-PCR. Moreover, the expression of four transcription factors related to anthocyanin biosynthesis was significantly down-regulated, namely *VubHLH92* (c112979), *VuMYB6* (c99078), *VuMYBPA1* (c115051), and *VuMYB12* (c117353). The expression of only *VuMYB6* and *VuMYBPA1* increased rapidly in ripening fruit, among the six different developmental stages of *V. uliginosum*, consistent with the accumulation of anthocyanin, whereas the expression of other transcription factors did not increase during the anthocyanin accumulation period, indicating they had no direct relationship with anthocyanin synthesis. *VuMYBB6* was annotated to *MYB1* by BLAST sequence alignment analysis, and *MYB1* and *MYBPA1* were found to both be R2R3-MYB type transcription factors. The significant down-regulation of the expression of *VuMYB1* and *VuMYBPA1* inhibited the expression of structural genes *VuCHS*, *VuFHT*, *VuF3’H*, *VuDFR*, *VuANS*, and *VuUFGT*, affecting the anthocyanin accumulation in the white-fruited variant. This study provided another possibility for the mechanism of mutation in *V. uliginosum*. We intend to verify the specific regulatory mechanism in the future.

## Conclusions

According to the transcriptome analysis of DEGs related to anthocyanin synthesis pahway in the ripening fruits of *V. uliginosum* and white-fruited variant, the down-regulated expression of transcription factors *VubHLH92*, *VuMYB6*, *VuMYB12*, and *VuMYBPA1* might lead to the down-regulation of the structural genes related to the anthocyanin synthesis pathway.

The results of the expression of these four transcription factors in different developmental stages of*V. uliginosum* showed that only the expression of *VuMYB6* and *VuMYBPA1* is related to the accumulation of anthocyanins.

*VuMYB6* was annotated as *MYB1* by the BLAST tool. Therefore, the down-regulation of the transcription factors *VuMYB1* and *VuMYBPA1* leads to the down-regulation of the structural genes associated with the anthocyanin synthesis pathway, which is the main reason for the lack of anthocyanins in the white berries.

## Materials and methods

### Plant materials

The ripening fruits (60 days after full-bloom stage) of *V. uliginosum* and its variant for RNA-seq were collected from Lanjia forest farm, Wangqing Contry, Jilin Province, China, and stored at − 80 °C.

Different developmental stages of *V. uliginosum* materials for q-PCR were also collected: blooming flowers (LS1), fruits with prominent ovarian enlargement 15 days after full-bloom stage (LS2), 0.5—0.7 cm fruits 30 days after full-bloom stage (LS3), 0.7—1.0 cm fruits 40 days after full-bloom stage (LS4), fruits at the stage when color changes 50 days after full-bloom stage (LS5), and the ripening fruits 60 days after full-bloom stage (LS6).

## Methods

### Extraction and detection of total RNA

The total RNA was isolated from the ripening fruits of *V. uliginosum* and its variant by using the RNA rapid extraction kit (Hua Yueyang, Beijing, China). Spectrophotometry method was used to detect the purity, concentration and integrity of RNA samples to ensure that the high-quality samples were used for RNA-seq.

RNA was also isolated from the materials collected at different developmental stages of *V. uliginosum* by using the RNA rapid extraction kit. The first chain of cDNA was synthesized using a reverse transcription Kit (Takara, Dalian, China) for q-PCR.

### cDNA library, deep transcriptome sequencing and assembly

Enrichment of *V. uliginosum* and its variant mRNA with magnetic beads with Oligo (dT), randomly interrupt mRNA by adding Fragmentation Buffer. With the mRNA from the fruits *V. uliginosum* and its variant as template, the first cDNA strand was synthesized using random hexamers, then a second cDNA strand was synthesized by adding buffer, dNTPs, RNase H and DNA polymerase I, and cDNA was purified using AMPure XP beads. The end was trimmed and a poly (A) tail was added at the sequencing joint. The AMPure XP beads were used to select the fragment size, and the cDNA library was obtained using PCR enrichment.

After the library was constructed, the concentration and insert size of the library were detected using Qubit2.0 and Agilent 2100 respectively, and the effective concentration of the library was accurately quantified by q-PCR method to ensure the library quality. Based on Sequencing By Synthesis technology, deep transcriptome sequencing was performed using Illumina HiSeq 2500 (Illumina, USA) and the read length was PE125. The Raw Data were filtered by removing the connector sequence and low-quality reads to achieve high-quality clean Data. Sequence alignment was conducted between the clean data for each sample and assembled transcript or unigene library. The obtained transcript and unigene reads were called Mapped Reads and were used for subsequent analysis. The two samples of *V. uliginosum* and its white-fruited variant were sequenced three times in order to ensure the accuracy of the experiment.

### Functional annotation of unigenes

The BLAST tool [33] was used to compare the unigene sequences with those in NR [34], Swiss-Prot [35], GO [36], COG [37], KOG [38], and KEGG [39] databases. The results of unigene orthology in KEGG were obtained using KOBAS 2.0 [40]. After the amino acid sequences of the unigenes were predicted, the unigenes were annotated by comparing with HMMER [41] by using the Pfam [42] database.

### Expression calculation of unigenes

The reads of each sequenced sample were compared with the unigene database by using Bowtie [43]. Based on this comparison we estimated the expression levels by using RSEM [44]. We used the value of FPKM to indicate the expression abundance of unigenes.

FPKM can eliminate the effect of gene length and sequencing on the calculation of gene expression. The calculated gene expression can be directly used to compare the differences in gene expression between different samples. The formula is as follows:

$$ \mathrm{FPKM}=\frac{\mathrm{cDNA}\kern0.17em \mathrm{Fragment}}{\mathrm{Mapped}\kern0.17em \mathrm{Fragment}\left(\mathrm{Mollions}\right)\ \mathrm{x}\ \mathrm{Transcript}\ \mathrm{Length}\left(\mathrm{kb}\right)} $$

### Differential gene expression and anthocyanin synthesis-related gene analysis

The FDR of less than 0.01 and the difference in expression level (fold change; FC) of ≥2 were used as a screening criterion. FC represents the ratio of the expression levels for two samples (groups). Therefore, FC > 2 indicates differences in the expression level of the two samples.

The DEGs and anthocyanin synthesis pathway and glucose metabolism-related genes expression in the ripening fruits of *V. uliginosum* and its white-fruited variant were analyzed using quantitative real-time polymerase chain reaction (qRT-PCR). Specific primers were designed and synthesized according to the gene sequence obtained using high throughput sequencing. Futher, specific primers of anthocyanin biosynthesis-related genes designed by Michael Zifkin [45] for *Vaccinium corymbosum* were used to verify the expression levels of the genes by using q-PCR (Additional file 5: Table S5). The *VuGAPDH* gene [27] (GenBank Accession No. KP218509) was as reference gene. The SYBR FAST qPCR Kit Master Mix (2×) and the universal dye method (KAPA Biosystems, USA) were used to validate the expression of the genes by using ABI7900HT real time quantitative PCR instrument (ABI Company, USA). The PCR condition was as follows: pre-incμbation at 95 °C for 5 min; amplification at 95 °C for 3 s, 60°C for 20 s and 95 °C for 15 s. The melting cμrves were measured at 60 °C for 15 s, 95 °C for 15 s. The genes expression levels of the ripening fruits of the white-fruited variant were compared with those of the blue ripening fruits of *V. uliginosum.* Data analysis was performed using the 2 ^-△△CT^ method. Statistical analysis was performed using *t*-test at the level of *P* ≤ 0. 05.

### Differential expression of transcription factors at the different developmental stages of *V. uliginosum*

The expression of significantly down-regulated transcription factors at the different developmental stages of the white-fruited variety of *V. uliginosum* was detected by performing q-PCR by using the 2 ^-△△CT^ method. The PCR condition was the same as mentioned abrove. Moreover, the gene expression level and anthocyanin accumulation in the samples were assessed using *VuGAPDH* as the reference gene. The expression of transcription factors at the different developmental stages of *V. uliginosum* was detected and compared with that at the full-bloom stage (LS1). Statistical analysis is performed using Duncan test at the level of *P* ≤ 0. 05. Each gene from each sample was analyzed three times to ensure the accuracy of the experimental results.

## Additional files

Additional file 1: **Table S1.** The up-regulated expression genes from the annotated DEGs (XLS 48 kb)

Additional file 2: **Table S2.** The down-regulated expression genes from the annotated DEGs (XLS 182 kb)

Additional file 3: **Table S3.** Differentially expressed genes identified using GO function enrichment (XLS 37 kb)

Additional file 4: **Table S4.** Differentially expressed genes from KEGG enrichment (XLSX 13 kb)

Additional file 5: **Table S5.** Specific primers used for anthocyanin biosynthesis pathway related genes (XLSX 13 kb)

## Abbreviations

*ANR*: Anthocyanidin reductase
*ANS*: Anthocyanidin synthase
*CCR*: Cinnamoyl-CoA reductase
*CHI*: Chalcone isomerase
*CHS*: Chalcone synthase
DEG: Differentially expressed genes
*DFR*: Dihydroflavonol 4-reductase
*F3’5’H*: Flavonoid3’, 5’hydroxylase
*F3’H*: Flavonoid 3′-hydroxylase
*F3H*: Flavanone 3-hydroxylase
FC: Fold change
FDR: False discovery Rate
*FHT*: Flavanone-3β-hydroxylase
GAPDH: Glyceraldehyde-3-phosphate dehydrogenase
*LAR*: Leucoanthocyanins reductase
MBW: MYB bHLH WD40
OPC: Procyanidolicoligomer
PAs: Proanthocyanidins
*POD*: Peroxidase
PPC: Procyanidolic polymer
q-PCR: quantitative polymerase chain reaction
qRT-PCR: quantitative real-time polymerase chain reaction
*UFGT*: UDP glucose-flavonoid 3-O-glucosyltransferase
